# Severe Congenital Neutropenia in a Newborn Caused by a Novel Mutation in the *ELANE* Gene—First Report From North Macedonia and a Literature Review: A Case Report

**DOI:** 10.1155/crpe/6873929

**Published:** 2025-12-08

**Authors:** Nikolina Zdraveska, Teodora Trajkovska, Aleksandra Jovanovska, Arjeta Hasani, Mica Kimovska Hristova

**Affiliations:** ^1^ Faculty of Medicine, University Children′s Hospital, Ss Cyril and Methodius University in Skopje, Skopje, North Macedonia, bg.ac.rs; ^2^ Department for Neonatology, Faculty of Medicine, University Children’s Hospital, Ss Cyril and Methodius University in Skopje, Skopje, North Macedonia, tirsova.rs; ^3^ Department for Pediatric Hematology and Oncology, Faculty of Medicine, University Children’s Hospital, Ss Cyril and Methodius University in Skopje, Skopje, North Macedonia, tirsova.rs; ^4^ Department for Immunology, Faculty of Medicine, University Children′s Hospital, Ss Cyril and Methodius University in Skopje, Skopje, North Macedonia, bg.ac.rs

**Keywords:** *ELANE* mutations, granulocyte colony-stimulating factor (G-CSF), neonatal neutropenia, neutrophil elastase, severe congenital neutropenia

## Abstract

**Background:**

Severe congenital neutropenia (SCN) is a rare group of hematologic disorders characterized by defects in the maturation and differentiation of neutrophilic granulocytes. This condition leads to severe chronic neutropenia, defined as an absolute neutrophil count of less than 0.5 × 10^9/L, and susceptibility to lethal pyogenic and fungal infections. SCN has a diverse genetic basis; however, it is most commonly linked to mutations in the *ELANE* gene, with over 230 mutations reported to date.

**Case presentation:**

We present a male term newborn who exhibited symptoms of neonatal pneumonia, delayed separation of the umbilical cord stump, and persistent neutropenia. Diagnosis was confirmed through bone marrow aspiration biopsy and a genetic analysis revealing the novel mutation c.200C > G (p.Ser67Trp) in the *ELANE* gene, marking the first reported case of SCN from North Macedonia. Additionally, a comprehensive review of published cases will be provided.

**Conclusion:**

SCN is rarely reported in newborns, and the diagnosis is often delayed or missed. Maintaining a high level of suspicion and ensuring early referral for genetic testing is required to reduce the risk of infection and improve the overall prognosis. Also, reporting cases harboring novel mutations is crucial for advancing our understanding of the disease pathogenesis and establishing phenotype–genotype correlations.

## 1. Introduction

Neutropenia presents an intriguing condition characterized by an absolute neutrophil count (ANC) below the established normal reference range for the corresponding age. In neonates younger than 10 days, ANC can vary considerably and is primarily influenced by the gestational age. The most common causes of neonatal neutropenia include infection‐induced neutropenia, immune‐mediated neutropenia, and transient neutropenia of infancy. Severe congenital neutropenia (SCN) represents a rare group of inherited disorders, with an estimated prevalence of 3–8.5 cases per million people, in which the impaired differentiation of neutrophilic granulocytes results in severe chronic neutropenia, with an ANC < 0.5 × 10^9/L [1]. SCN is a genetically heterogeneous disorder with more than 30 genes implicated. The most common subtype is the autosomal dominant form, linked to mutations in the *ELANE* gene. This gene encodes neutrophil elastase, a serine protease essential for neutrophil differentiation and function, and more than 230 variants have been documented to date. Several pathophysiological mechanisms have been proposed to explain congenital neutropenia associated with *ELANE* mutations. The most widely accepted suggests that misfolded neutrophil elastase triggers endoplasmic reticulum stress and activation of the unfolded protein response, resulting in the apoptosis of promyelocytes during granulopoiesis [2]. Mutations in the *ELANE* gene cause congenital neutropenia, clinically differentiated into two forms: cyclic neutropenia (CyN) and SCN, depending on whether the reduction in neutrophil granulocytes in the peripheral blood occurs intermittently or persists continuously. While the mutation patterns in SCN and CyN typically differ, there is significant mutation overlap in both forms [3].

Although SCN is a congenital disorder, due to the low incidence and clinical heterogeneity, it is rarely described in newborns, often leading to delayed or missed diagnoses. In this report, we present a case of a newborn male diagnosed with SCN caused by a novel mutation in the *ELANE* gene, followed by a comprehensive review of published cases. To our knowledge, this is the first reported case of SCN from Macedonia.

## 2. Case Presentation

А 2‐day‐old male neonate was admitted to the neonatology department due to significantly elevated inflammatory markers, raising concern for neonatal sepsis. The newborn was delivered at 40 weeks of gestation, weighing 3620 g. The Apgar scores were 8 and 9 at the first and fifth minutes. He was the first child of healthy, young, nonconsanguineous parents of Macedonian origin. The mother received routine antenatal care and tested negative for GBS. A few hours after birth, due to signs of respiratory distress, noninvasive oxygen support was administered, and empirical antibiotic treatment was initiated with ampicillin and gentamycin, according to the neonatal early‐sepsis protocols. By the second postnatal day, the baby remained stable with minimal oxygen support. A comprehensive laboratory and microbiological workup was conducted, revealing normal hemoglobin levels and RBC count, leukopenia (WBC 4.05 × 10^9/μl), and significant neutropenia (ANC 0.01 × 10^9/μl). The inflammatory markers were elevated: CRP, 92.5 mg/L (normal < 5 mg/L), and procalcitonin, 53.9 ng/mL (normal < 0.5 ng/mL for the corresponding postnatal age). Thus, the antibiotic regimen was revised to include meropenem and amikacin, as well as local ciprofloxacin, due to blepharitis. *Escherichia coli* and *Enterococcus* species were cultured from the pharyngeal and eye swabs, respectively. The blood culture was negative. The peripheral blood smear revealed profound neutropenia, accompanied by an increase in monocytes and lymphocytes.

Immunological evaluation showed IgG 9.1 g/L (reference range 3.99–14.8 g/L), IgA 0.25 g/L (reference: 0.09–0.4 g/L), and IgM 0.72 g/L (reference: 0.05–0.7 g/L). Flow cytometric analysis showed normal lymphocyte subpopulations (CD3+ 82.4%, CD4+ 32.3%, CD8+ 21.8%, and CD3+/16 + 56+ (NK cells) 0.3%).

Ultrasound screening of the heart, abdomen, and urinary tract revealed no associated congenital abnormalities. There was notable clinical improvement over the following days; however, a concern arose regarding delayed separation of the umbilical cord stump, with erythema surrounding the umbilicus. Thus, local antibiotics as well as surgical ligation were provided.

**Figure 1:**
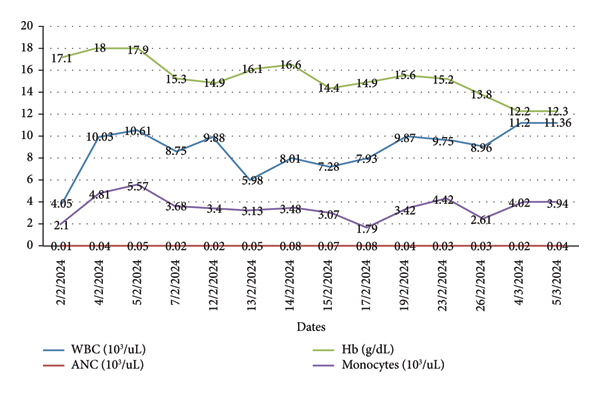
Values of white blood cells (WBCs), absolute neutrophil counts (ANCs), monocytes, and hemoglobin (Hb) testing at different times.

**Figure 2:**
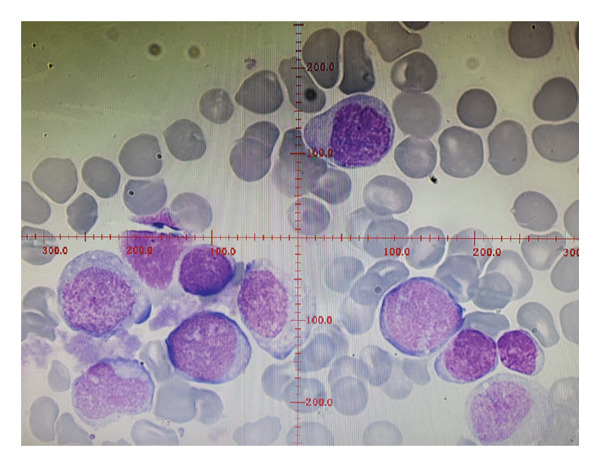
Bone marrow cytology. Granulopoiesis consisted predominantly of promyelocytes and myelocytes in maturation arrest. No excess blasts were noted.

Serial monitoring of complete blood count (Figure 1) revealed persistent neutropenia, ANC ranging from 0.01 to 0.08 × 10^9/μl, and monocytosis. To clarify the diagnosis, a bone marrow aspiration was performed at 3 weeks of age, showing maturation arrest of myeloid cells at the myelocyte stage. No excess blasts were noted on morphology (Figure 2).

The diagnosis of SCN was confirmed with a heterozygous mutation c. 200C > G in exon 2 of the *ELANE* gene, resulting in an amino acid change from serine to tryptophan at position 67 (p.Ser67Trp). This mutation was not previously reported but is classified as pathogenic according to the American College of Medical Genetics criteria. In the patient, the mutation was de novo, as it was not present in the parents. No pathogenic variants were detected in other genes associated with SCN (*HAX1, CSF3R, G6PC3*, *GFI1, VPS45*, and *WAS).*

The baby was discharged on the 34th postnatal day, clinically stable with no active infection, and was advised to continue with oral antibiotics and antifungal prophylaxis.

Regular checkups were performed at the neonatal outpatient clinic, including routine blood count monitoring. Persistent neutropenia with monocytosis was consistently noted.

**Figure 3:**
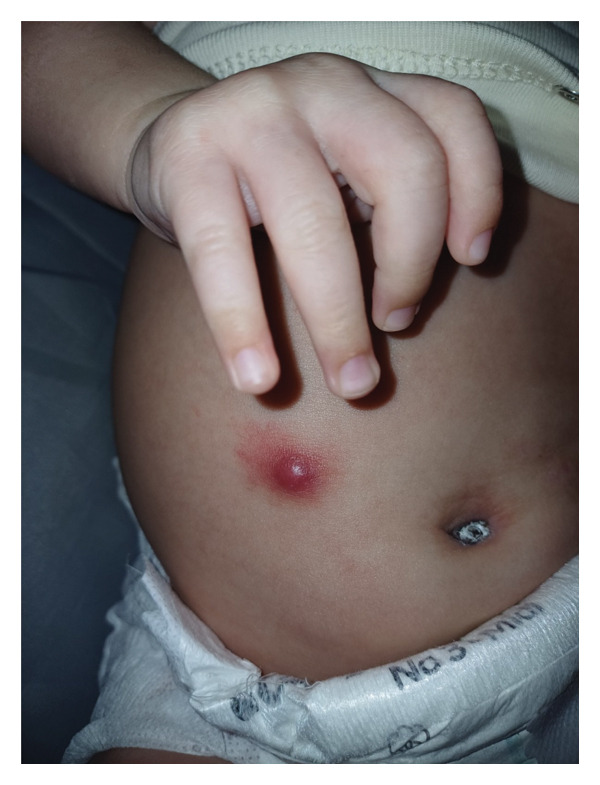
A reddish, tender, and swollen furuncle at the anterior abdominal wall.

At the age of 3 months, the infant presented with a localized, inflamed skin lesion on the anterior abdominal wall, which evolved into a painless, indurated lesion resembling a furuncle (Figure 3), measuring 2.5 cm in width, without purulent discharge. Consequently, the infant was readmitted, and parenteral antibiotic treatment was provided. The lesion regressed and was completely resolved after treatment.

Prophylactic antibiotic and antifungal therapy, consisting of sulfamethoxazole/trimethoprim and nystatin, was administered regularly by the parents, and no serious bacterial infections were noted during subsequent follow‐up. The infant exhibited normal growth and development. Treatment with granulocyte colony‐stimulating factor (G‐CSF) was initiated at approximately 1 year of age, with a starting dose of 5 μg/kg/day, which was gradually increased. The 6‐month follow‐up period since the introduction of G‐CSF therapy resulted in a mild, sustained increase in the ANC up to 0.8 × 10^9/μl.

## 3. Discussion

We have conducted a comprehensive search of the published literature in the PubMed and Embase databases to identify relevant articles in the English language. Various combinations of search terms were used, such as “congenital neutropenia,” “severe congenital neutropenia,” and “*ELANE* mutation.” A total of 132 articles were retrieved, including 28 case reports. The reference lists of the chosen papers were then screened for identifying additional relevant publications. Studies with missing detailed clinical data or duplicate cases were excluded from the analysis.

The clinical and molecular characteristics of 27 patients, including six familial cases, diagnosed with SCN due to mutations in the *ELANE* gene, are summarized in Table 1. The patients demonstrate a diverse range of backgrounds: Chinese, *n* = 8 [4–9]; Korean, *n* = 4 [17–20]; American, *n* = 3 [24–26]; Indian, *n* = 3 [12–14]; Mexican, *n* = 2 [21]; French, *n* = 1 [10]; Greek, *n* = 1 [11]; Japanese, *n* = 1 [16]; Italian, *n* = 1 [15]; Swedish, *n* = 1 [22] Tanzanian, *n* = 1 [23]; and Vietnamese, *n* = 1 [27]. The gender distribution was nearly equal, with 13 males and 14 females among the patients.

**Table 1:** Cases with severe congenital neutropenia caused by the *ELANE* mutation.

#	Gender/age	Country	Clinical presentation	Mutation	Treatment	Reference
1.	Female, 22 months	China	Recurrent infections (respiratory tract infection, otitis media, lymphadenitis), poorly responsive to antimicrobial therapy Autoimmune hemolytic anemia	c.452G > T (p.C151F)	Allogeneic hematopoietic stem cell transplantation (HSCT)	[4]
2.	Male, 22 months	China	Intermittent fever and cough Past history: fever and omphalitis in the neonatal period; cyclic neutropenia and ANCA‐associated vasculitis.	c.242G > C (p.R81P)	G‐CSF (when ANC < 0.5 × 10^9^/L) Positive response	[4]
3.	Male, 15 months	China	Persistent neutropenia since the neonatal period with recurrent infections	c.170 C > A (p.Ala57Asp)	Antibiotics G‐CSF refused by the family	[5]
4.	Male, 6 months	China	Persistent symptoms of recurrent fever and prolonged neutropenia lasting over 5 months	c.295_303del	G‐CSF (2–5 μg/kg/day for 3–5 days) improved ANC	[6]
5.	Male, 2 years 9 months	China	Prolonged fever Past history: recurrent respiratory infections after 1 year of age, mycotic stomatitis, perianal abscess, pneumonia	c.290A > C Coexistence of mutation in GFI1 c.298 + 28_68del40	Antibiotics, Antitubercular treatment	[7]
6.	Female, 10 months, Patient 1	China	Recurrent infections (monthly), stomatitis, cervical suppurative lymphadenitis, and pneumonia	c. 302T > G (p. V101G)	Antibiotics, HSCT	[8]
7.	Female, 4 years, Patient 2	China	Recurrent pyoderma, pneumonia, cervical lymphadenitis, and chronic gingivitis since the age of 2 months	c. 302T > G (p. V101G)	Antibiotics, HSCT	[8]
8.	Male, 2.5 years	China	Recurrent fever, skin and soft tissue infections Past history: at 14‐day age: neonatal septicemia, purulent meningitis, skin infection	c.125 C > T (p.P42L)	Antibiotics, G‐CSF	[9]
9.	Female, adult	France	Multiple severe infections and recurrent gingivitis	c.242G > C p.Arg81Pro	Continuous G‐CSF treatment from 6 months of age	[10]
10.	Female, 5 years	Greece	Recurrent skin and respiratory infections Past history: skin abscess (40 days), lobar pneumonia (5 years)	c.157C > G (p.His53Asp)	Antibiotics, G‐CSF	[11]
11.	Male, 2 months	India	Omphalitis, hepatic hemangioendothelioma	c.215T > A, (p.Val72Glu)	Antibiotics, G‐CSF	[12]
12.	Female, 3 years	India	Recurrent skin and upper respiratory tract infection	c.201T > A	Antibiotics, G‐CSF	[13]
13.	Male, 20 months	India	Pneumonia and otitis media (2 months), skin ulcer	c.457G > C (p.Ala153Pro)	Antibiotics, G‐CSF	[14]
14.	Female, 4 years	Italy	Frequent febrile episodes, skin abscesses, bronchopneumonia myelodysplastic syndrome, long‐lasting invasive pulmonary mycosis (8 years); acute myeloid leukemia	Multiple mutations c.2192G > A c. 2240G > A IVS3‐22G > A	Antibiotics, G‐CSF, HSCT (8 years)	[15]
15.	Female, 1 year	Japan	Recurrent serious skin infections Acute myeloid leukemia (8 years)	c.1A > G (p. Met1Val)	G‐CSF (up to 40 μg/kg) HSCT (8 years)	[16]
16.	Female, 9 months	Korea	Recurrent cervical lymphadenitis without abscess formation. History of omphalitis and isolated neutropenia at birth.	c.607G > C (p.Gly203Arg)	IV antibiotics, G‐CSF (15 μg/kg/d)—temporary response	[17]
17.	Male, 20 years	Korea	Fever and knee abrasions after an accident Profound neutropenia in infancy Latent tuberculosis infection at 15 years	c597+1G > C (pV190‐F199del)	Monitored closely for infection, minimum use of granulocyte colony‐stimulating factor (G‐CSF).	[18]
18.	Male, 1.5 months	Korea	Otitis media Older sister died from pneumonia (2 years)	c.658delC (p.Arg220Glyfs20) somatic mosaicism in the father	G‐CSF, antibiotics	[19]
19.	Female, 17 months	Korea	Cervical lymphadenitis (MRSA), sepsis, and peritonitis with perforated appendicitis (8 months) Sister (3 years) and mother (32 years) with recurrent stomatitis	c.597+1G > A same mutation in older sister and mother	Antibiotics, G‐CSF, surgical excision	[20]
20.	Female, newborn, Patient 1	Mexico	Neonatal sepsis. At 45 days, had soft tissues infections of right nostril and umbilical scar.	c.607G > C (p.Gly203Arg)	Treated with G‐CSF. Good clinical response without complications.	[21]
21.	Female, Patient 2	Mexico	At 16 days of age hospitalized for omphalitis and neonatal sepsis	c.607G > C (p.Gly203Arg)	Treated with G‐CSF. Good clinical response without complications.	[21]
22.	Male, 3 months	Sweden	Periorbital bacterial cellulitis (newborn) Otitis media, umbilical granuloma (2 months)	Double mutations c.90T > G (p.Ile30Met) c.194T > A (p.Val65Asp)	Antibiotics, G‐CSF (up to 30 μg/kg)	[22]
23.	Male, 1 year	Tanzania	Right‐sided cervical lymphadenopathy Multiple skin abscesses and sepsis Recurrent fever, fungal pneumonia (2.5 years)	c.234–242del9	Antibiotics, G‐CSF, HSCT (3 years)	[23]
24.	Male, newborn	USA	Omphalitis, recurrent bacterial infection	c.356T > A (p.V119E)	High doses of G‐CSF required; HSCT at the age of 7 months; on Day +65, second transplant	[24]
25.	Female, 2 years	USA	Otitis media, cellulitis	p.G214 V	G‐CSF, antibiotics	[25]
26.	Female, newborn	USA	Anemia and jaundice (ABO incompatibility) Persistent neutropenia without signs of infection	c.15862C > T (p.Pro110Leu)	G‐CSF	[26]
27.	Male, 6 years	Vietnam	Recurrent infections (misdiagnosed with tuberculosis and autoimmune neutropenia) Necrotizing pneumonia (1.5 years); retroauricular abscess (6 years)	c.242G > C	Antibiotics, abscess drainage, right partial lobectomy, G‐CSF	[27]

The clinical manifestations varied from mild infections to life‐threatening conditions in the cases analyzed. The most prevalent complaint was recurrent or prolonged fever episodes, which occurred in 66.7% of cases. Other significant symptoms included mucosal ulcers or gingivitis in 29.6% of the cases, cervical adenopathy in 26%, and otitis in 22%. In the majority of analyzed cases (16 out of 27), the diagnosis was made after infancy, a period when recurrent infections become more frequent. The emergence of serious infections at a later age, such as pneumonia and deep abscesses, served as indicators for diagnosing SCN in nearly one‐third of the analyzed cases.

Identifying SCN during the neonatal period, as illustrated in our case, can be particularly challenging. This condition presents an even greater diagnostic obstacle in resource‐constrained countries where access to advanced genetic technologies may be restricted; therefore, high suspicion and early bone marrow analysis may facilitate prompt diagnosis. In neonates with SCN, the most common clinical sign observed was omphalitis, occurring in 85.7% of the cases.

Our patient presented with neonatal pneumonia caused by *E. coli*, which is a serious condition in newborns. Initially, the neutropenia was considered infection‐related, as this is the most common form of neutropenia in infants. Typically, the duration of neutropenia is short, lasting less than 10 days; however, some infections can result in prolonged neutropenia. Notably, our patient did not exhibit any signs of omphalitis, and following clinical improvement, the only remaining concern was the delayed separation of the umbilical cord stump.

Given the lack of a specific phenotype indicative of particular syndromes, we adhered to the European guidelines for differentiating neonatal neutropenia [1]. After defining “true neutropenia” with ANC outside the normal range for the corresponding gestational and postnatal age, other contributing factors such as prenatal growth retardation, asphyxia, Rh hemolytic disease of the newborn, maternal hypertension, and exposure to antiretroviral therapy or chemotherapy were excluded. The only factor retained for consideration was maternal tobacco smoking, though it typically affects all leukocyte subsets and is not persistent. In early infancy, our patient developed a skin abscess without pus, one of the defining clinical characteristics of SCN.

The current patient is the first Macedonian child diagnosed with SCN to undergo a thorough investigation, during which the underlying mutation was identified. Molecular analysis revealed a novel missense mutation, c.200C > G (p.Ser67Trp), located in exon 2 of the *ELANE* gene, causing a structural protein change. This mutation is classified as pathogenic, as it meets the criteria for classifying pathogenic variants (PS2, PM1, PM2, PP3, PP4, and PP5) [28].

In the analyzed cohort of 27 patients, 21 distinct mutations were identified. The c.607G > C was reported in three analyzed patients: two familial cases (#20 and #21) and a nonrelated Korean girl (#16), all of whom were diagnosed with SCN during infancy [17, 21]. Another frequently reported mutation c.242G > C was identified in three unrelated patients with diverse ancestries, who were diagnosed with SCN (#2, #9) and CyN (#27), highlighting the genetic overlap between the two conditions [4, 10, 27].

Although rarely, digenic (#5) and multiple mutations (#14 and #22) were described in some patients [7, 15, 22]. While the authors could not determine any interactions between the digenic variants in the reported patient (#5), there are examples in various clinical disorders where digenic mutations can amplify the phenotype associated with a primary mutation. In addition, the presence of multiple *ELANE* mutations was associated with poor clinical course, requiring high‐dose G‐CSF treatment and malignant transformation, suggesting the potential negative synergistic effect.

The management of SCN involves the prevention of infections with antimicrobial and antifungal prophylaxis. In addition to anti‐infective treatment, the long‐term management of SCN typically requires recombinant human G‐CSF, which effectively increases neutrophil counts. The required dosage of G‐CSF varies widely, starting at 5 μg/kg/day, administered subcutaneously daily, and gradually increasing every 3–5 days until a rise in the ANC is observed. When the G‐CSF requirements exceed 50 μg/kg/day, patients are considered nonresponsive and may be candidates for stem cell transplantation [29].

In our case, we postponed the G‐CSF therapy until 1 year of age, despite persistently low neutrophil counts due to the absence of serious infections, and given the possible adverse effects of the long‐term treatment. Since this mutation has not been previously described, the patient’s response to G‐CSF and the potential complications will be closely monitored.

Among the patients analyzed, 81% received G‐CSF treatment, and most showed positive responses, characterized by increased white blood cell counts and a reduction in infections (17 out of 22 patients, or 77%). Hematopoietic stem cell transplant treatment was provided in five patients due to invasive infections and poor response to G‐CSF, and development of malignancy (acute myeloid leukemia [AML], *n* = 2). Previously, the cumulative incidence of disease progression to myelodysplastic syndrome and/or AML was reported to be 13% after 8 years of G‐CSF treatment and up to 22% and 31% after 15 years of G‐CSF treatment, according to data from the Severe Chronic Neutropenia International Registry [30].

Beyond supportive care, emerging genetic approaches highlight the potential of CRISPR/Cas9 gene therapy for *ELANE* mutations in SCN. Nasri et al. recently presented a successful and efficient strategy for inhibiting *ELANE* mRNA expression by targeting the regulatory region in the promoter located upstream of the transcription start site of the *ELANE* gene. This resulted in effective granulocytic differentiation of the edited cells, both in vitro and in vivo, and holds a promising potential for effective gene therapy in patients with *ELANE*‐related SCN [31].

In conclusion, we present an *ELANE* gene mutation‐associated SCN in a newborn boy from Macedonia. The novel *ELANE* mutation may contribute to the accumulation of SCN cases, aiming to establish phenotype–genotype correlations. A high level of suspicion is required for the timely diagnosis of affected patients, as this can reduce the risk of infection and improve the overall prognosis.

## Ethics Statement

Ethics committee approval was received from the Medical Faculty Skopje Ethics Committee (Ref. number 05‐2210/1; 07.04.2025).

## Consent

Written informed consent was obtained from the patient’s parent to publish this report in accordance with the journal’s patient consent policy.

## Conflicts of Interest

The authors declare no conflicts of interest.

## Funding

The authors received no specific funding for this work.

## Data Availability

The data that support the findings of this study are available from the corresponding author upon reasonable request.
